# Predictive molecular biomarkers for determining neoadjuvant chemosensitivity in muscle invasive bladder cancer

**DOI:** 10.18632/oncotarget.28302

**Published:** 2022-11-02

**Authors:** Neal Murphy, Andrew J. Shih, Paras Shah, Oksana Yaskiv, Houman Khalili, Anthony Liew, Annette T. Lee, Xin-Hua Zhu

**Affiliations:** ^1^Donald and Barbara Zucker School of Medicine at Hofstra/Northwell, Hempstead, NY 11549, USA; ^2^Northwell Health Cancer Institute, Lake Success, NY 11042, USA; ^3^Feinstein Institutes for Medical Research, Manhasset, NY 11030, USA; ^4^Mayo Clinic, Rochester, MN 55902, USA; ^5^Northwell Health Department of Pathology, Greenvale, NY 11548, USA; ^*^These authors contributed equally to this work and share first authorship; ^#^These authors contributed equally to this work and share last authorship

**Keywords:** muscle invasive bladder cancer, neoadjuvant chemotherapy, gene expression, molecular subtyping, canonical correlation analysis

## Abstract

Introduction: Identifying neoadjuvant chemotherapy (NAC) response in patients with muscle invasive bladder cancer (MIBC) has had limited success based on clinicopathological features and molecular subtyping. Identification of chemotherapy responsive cohorts would facilitate delivery to those most likely to benefit.

Objective: Develop a molecular signature that can identify MIBC NAC responders (R) and non-responders (NR) using a cohort of known NAC response phenotypes, and better understand differences in molecular pathways and subtype classifications between NAC R and NR.

Materials and Methods: Presented are the messenger RNA (mRNA) and microRNA (miRNA) differential expression profiles from initial transurethral resection of bladder tumor (TURBT) specimens of a discovery cohort of MIBC patients consisting of 7 known NAC R and 11 NR, and a validation cohort consisting of 3 R and 5 NR. Pathological response at time of cystectomy after NAC was used to classify initial TURBT specimens as R (pT0) versus NR (≥pT2). RNA and miRNA from FFPE blocks were sequenced using RNAseq and qPCR, respectively.

Results: The discovery cohort had 2309 genes, while the validation cohort had 602 genes and 13 miRNA differentially expressed between R and NR. Gene set enrichment analysis identified mitochondrial gene expression, DNA replication initiation, DNA unwinding in the R discovery cohort and positive regulation of vascular associated smooth muscle cell proliferation in the NR discovery cohort. Canonical correlation (CC) analysis was applied to differentiate R versus NR. 3 CCs (CC13, CC16, and CC17) had an AUC >0.65 in the discovery and validation dataset. Gene ontology enrichment showed CC13 as nucleoside triphosphate metabolic process, CC16 as cell cycle and cellular response to DNA damage, CC17 as DNA packaging complex. All patients were classified using established molecular subtypes: Baylor, UNC, CIT, Lund, MD Anderson, TCGA, and Consensus Class. The MD Anderson p53-like subtype, CIT MC4 subtype and Consensus Class stroma rich subtype had the strongest correlation with a NR phenotype, while no subtype had a strong correlation with the R phenotype.

Conclusions: Our results identify molecular signatures that can be used to differentiate MIBC NAC R versus NR, salient molecular pathway differences, and highlight the utility of molecular subtyping in relation to NAC response.

## INTRODUCTION

In 2022, the American Cancer Society estimates there will be 81,180 new cases of bladder cancer diagnosed in the United States and 17,100 people will die from the disease [1]. About half of these initial cases are superficial to the muscularis propria and have a 5-year overall survival of 95%, while about one third of these initial cases are found to be muscle invasive bladder cancer (MIBC) with a significant decrease in 5-year survival rate to 69% [2, 3]. For MIBC, neoadjuvant chemotherapy (NAC) prior to cystectomy is standard of care and provides a 5–8% increase in 5-year overall survival compared to cystectomy alone [4, 5]. In addition, patients who achieve a pathologic response to NAC have a 5-year survival rate of about 80–90% compared to 40–50% in non-responders [6]. However, a significant number of patients do not respond to NAC, with reported pT0 rates of 38% to neoadjuvant methotrexate, vinblastine, doxorubicin, and cisplatin (MVAC) and accelerated MVAC [6, 7], as well as pT0 rates of 42% to dose-dense MVAC (ddMVAC) and 36% to gemcitabine, cisplatin (GC) [8]. The NAC non-responders suffer from unnecessary adverse effects and a delay in time to cystectomy leading to worse overall survival [9, 10]. Subsequently, there remains a critical need to understand the molecular biology behind NAC responsiveness, in order to better tailor individual NAC therapy.

Gene expression profiling of MIBC holds promise for future individualization of therapy, and several studies have attempted to predict chemosensitivity using the molecular profile of tumors. Somatic mutations in the following DNA damage repair genes have been found to correlate with response to chemotherapy: ERCC2, ATM, RB1, and FANCC [11, 12]. This discovery has led to ongoing clinical trials designed to provide bladder sparing approaches after NAC using these biomarkers (NCT03609216). However, these mutations are only one part of the complex biological pathway driving bladder cancer, with <15% of patients harboring mutations in ATM and ERCC2 in the 2017 TCGA analysis [13]. Smith et al. used a microarray analysis to identify genes from the National Cancer Institute’s Developmental Therapeutics Program (NCI-DTP) whose expression was related to *in vitro* drug sensitivity, and then determined which of these genes maintained concordant expression with *in vitro* chemosensitivity analysis from 40 commonly used bladder cancer lines [14]. The SWOG S1314 Phase II study was designed to evaluate this approach, named Coexpression Extrapolation (COXEN), in order to determine if a treatment-specific COXEN score can predict NAC pathologic response [15]. Results from the trial indicated that the COXEN scores were not significantly prognostic for chemotherapy response in the individual treatment arms [16].

Moving one step further, transcriptomic profiling and unsupervised class discovery performed in several studies have found prognostic molecular subtypes [13, 17–23]. These classifications have been derived from non-overlapping datasets using different methods, which has generated conflicting results and no general consensus on a definitive subtype classification. Furthermore, none of the studies have attempted to develop a classification based on known NAC response, which in our study is defined as a pathological complete response pT0 for chemotherapy responders (R) and ≥pT2 for chemotherapy non-responders (NR).

Therefore, our MIBC patient population with its known chemotherapy response phenotype represents a unique cohort to understand both the molecular mechanisms driving NAC response and to identify a molecular signature that truly correlates with NAC response. Here we present the differential mRNA and miRNA expression analysis of a discovery cohort of known chemotherapy responders versus non-responders. Expression differences are used to identify NAC responsiveness, which was further validated in a validation cohort of eight patients, as well as identify relevant molecular pathways in NAC non-responders and responders, and examine the correlation between established molecular subtypes and NAC response.

## RESULTS

### Demographics

Patients from 2008–2018 diagnosed with MIBC on a transurethral resection of bladder tumor (TURBT) were selected from the Northwell Health tumor bank. Patients received either 4 cycles of neoadjuvant GC or 3 or more cycles of ddMVAC chemotherapy. Pathological response at time of cystectomy was used to classify initial TURBT specimens as NAC responders with pathological complete response (pT0) versus non-responders with muscle invasive bladder cancer (≥pT2).

A total of 26 patients with known NAC response were identified, and inclusion in either the discovery or validation cohort was chosen at random. The discovery cohort consisted of 18 patients: 11 NR and 7 R. The average age of the entire cohort was 65, with about half receiving ddMVAC and half receiving GC. The majority of the patients were male. The NR group had an average time to recurrence of 13 months. The validation cohort consisted of 8 patients: 5 NR and 3 R. The average age of the entire cohort was 67 with the majority being male patients (see Table 1).

**Table 1:** Patient demographics

Discovery Cohort					
Path Status	Gender	Age at TURBT	NAC	Cys Path Stage	Time to Recur (Months)
NR1	M	53	ddMVAC	pT3aN0	26.6
NR2	M	79	GC	pT4aN0	5.2
NR3	M	68	GCarbo	pT3bN0	28.6
NR4	M	69	N/A	pT3bN0	20
NR5	M	67	ddMVAC	pT3aN2	9
NR6	F	72	GC	pT2bN0	no recurrence
NR7	M	64	ddMVAC	pTisN1	4.9
NR8	F	68	GC	pT3N2	5.2
NR9	M	62	ddMVAC	pT3bN1	3.2
NR10	M	61	GC	pT2aN0	19
NR11	M	66	ddMVAC	pT3aN1	N/A
R1	M	64	GC	pTisN0	no recurrence
R2	F	56	GC	pT0N0	no recurrence
R3	F	57	ddMVAC	pT0N0	no recurrence
R4	M	59	N/A	pT0N0	no recurrence
R5	M	65	N/A	pT0N0	no recurrence
R6	F	70	N/A	pT0N0	no recurrence
R7	M	75	GC	pTisN0	no recurrence
**Validation Cohort**					
NR12	F	60	ddMVAC	pT3	9.1
NR13	M	52	N/A	pT2bN0	no recurrence
NR14	M	61	ddMVAC	pT2bN0	N/A
NR15	F	80	GC	pT2bN0	9.8
NR16	M	93	GC	pT2aN0	N/A
R8	M	66	ddMVAC	pT0N0	no recurrence
R9	M	67	GC	pT0N0	no recurrence
R10	F	57	GC	pT0N0	no recurrence

### RNAseq expression analysis

**Figure 1:**
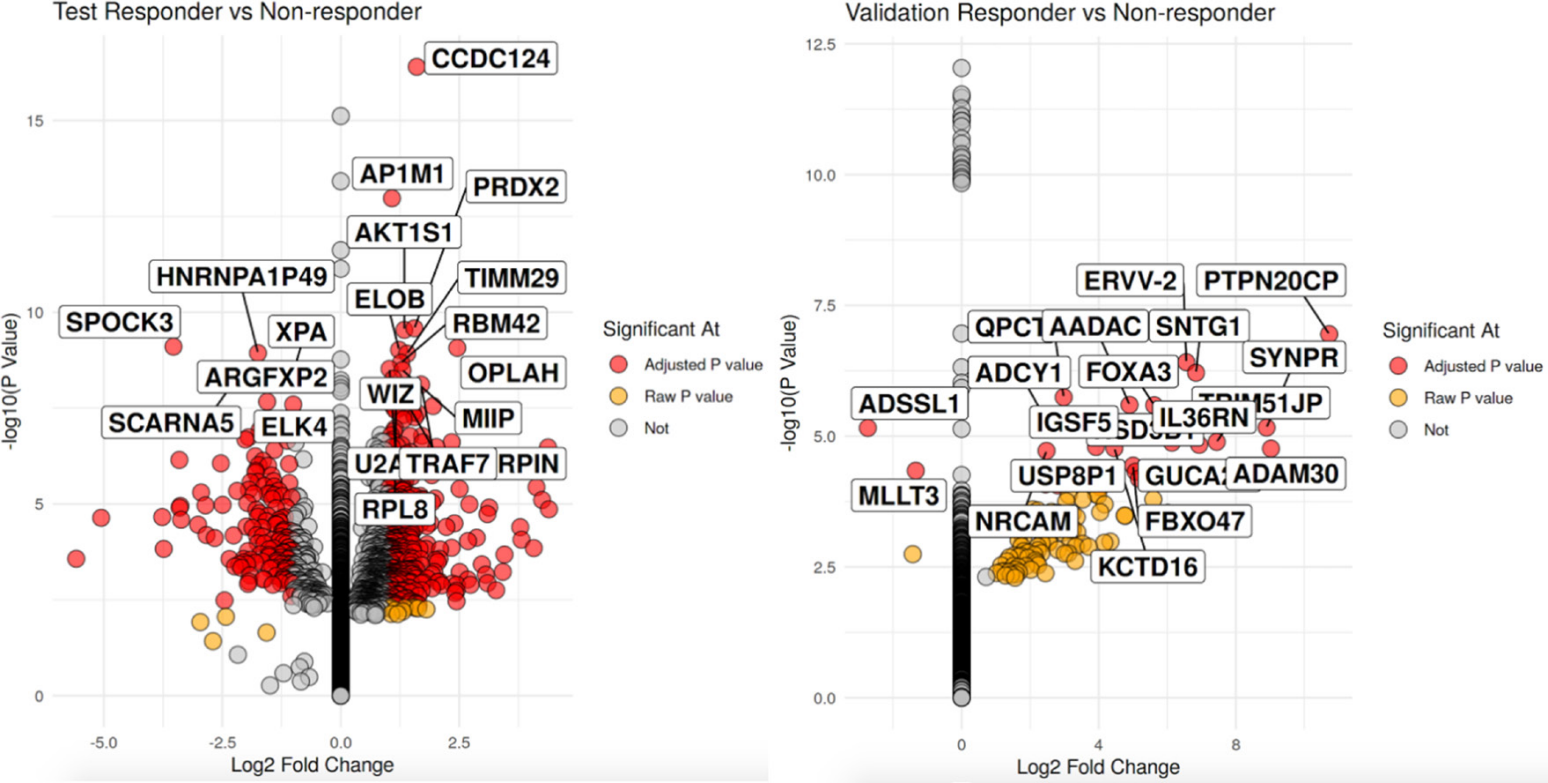
Volcano plot of significant differentially expressed genes with the left volcano being the discovery cohort and validation cohort on the right. For each volcano plot, R are on the left side nand NR on the right side pf each volcano. X-axis is the log 2 fold change between groups while the y-axis is the -log 10 raw *p*-value. The top 20 genes by *p*-value are labeled.

In the discovery cohort, 2039 genes had significant expression differences between the R vs. NR groups after accounting for multiple testing corrections (See Figure 1 for Top 20 genes in both cohorts and Supplementary Table 1 for the complete list of genes). Of those genes, a slight majority were seen in the R group (1,041 genes) compared to the NR group (998 genes). In the validation cohort, 602 genes had significant expression differences between the R vs. NR groups (*p*-value < 0.01, data included in Supplementary Table 2), with the majority seen in the R group (509 genes) compared to NR (93 genes).

Pathway enrichment analysis using GSEA (gene set enrichment analysis) identified 519 gene sets in the R discovery cohort (see Table 2 for top 12 biologically relevant gene sets for R and NR) and 155 gene sets in the R validation cohort with a *p*-value < 0.01. 33 of these gene sets overlapped. For the NR discovery cohort, there were 60 gene sets and 354 gene sets in the NR validation cohort with a *p*-value < 0.01. 18 of these gene sets overlapped (Supplementary Table 3).

**Table 2:** GSEA analysis, Top 12 pathways for the R discovery cohort on the top and NR discovery cohort on the bottom, based on *p*-value < 0.01

R	GOBP_Pathway
1	GOBP_KERATINIZATION
2	GOBP_KERATINOCYTE_DIFFERENTIATION
3	GOBP_SULFUR_COMPOUND_CATABOLIC_PROCESS
4	GOBP_DNA_DEPENDENT_DNA_REPLICATION
5	GOCC_ORGANELLAR_RIBOSOME
6	GOBP_ANTIMICROBIAL_HUMORAL_RESPONSE
7	GOCC_CORNIFIED_ENVELOPE
8	GOBP_DNA_REPLICATION_INITIATION
9	GOBP_MITOCHONDRIAL_TRANSLATION
10	GOCC_MITOCHONDRIAL_LARGE_RIBOSOMAL_SUBUNIT
11	GOBP_EPIDERMAL_CELL_DIFFERENTIATION
12	GOBP_DNA_UNWINDING_INVOLVED_IN_DNA_REPLICATION
**NR**	**GOBP_Pathway**
1	GOBP_GENE_SILENCING_BY_RNA
2	GOMF_RNA_BINDING_INVOLVED_IN_POSTTRANSCRIPTIONAL_GENE_SILENCING
3	GOBP_CELL_MIGRATION_INVOLVED_IN_SPROUTING_ANGIOGENESIS
4	GOBP_REGULATION_OF_SPROUTING_ANGIOGENESIS
5	GOBP_REGULATION_OF_CELL_MIGRATION_INVOLVED_IN_SPROUTING_ANGIOGENESIS
6	GOBP_BLOOD_VESSEL_ENDOTHELIAL_CELL_PROLIFERATION_INVOLVED_IN_SPROUTING_ANGIOGENESIS
7	GOBP_VASCULAR_ASSOCIATED_SMOOTH_MUSCLE_CELL_MIGRATION
8	GOBP_SMOOTH_MUSCLE_CELL_DIFFERENTIATION
9	GOBP_SPROUTING_ANGIOGENESIS
10	GOMF_EXTRACELLULAR_MATRIX_STRUCTURAL_CONSTITUENT_CONFERRING_TENSILE_STRENGTH
11	HP_SPARSE_BODY_HAIR
12	GOMF_METALLOENDOPEPTIDASE_ACTIVITY

### miRNA expression analysis

In the discovery cohort, no miRNA were found to meet statistical significance (Supplementary Table 4), however the validation cohort had 13 miRNAs that met a *p*-value threshold of .05. miR-18a was upregulated in the R group while the remaining 12 were seen in the NR group (Supplementary Table 5).

### Canonical correlation analysis in the discovery and validation cohorts

Canonical correlation analysis (CCA) is used to determine and measure the association amongst two different sets of variables and redefine them as canonical components (CCs). Whereas Principal Component Analysis (PCA) focuses on finding linear combinations that contribute to the most variance in a data set, CCA focuses on finding linear combinations that correlate the most between two datasets. In essence, CCs are *de novo* combinations of genes and miRNA that have correlated expression patterns, and each CC can be seen as a co-expression network with its own molecular profile with the potential to influence a clinical outcome.

A total of 17 CCs were identified (Supplementary Table 6). To classify patients as R versus NR, univariate logistic regression was performed on the CCs. 3 CCs (CC13, CC16, and CC17) have an AUC >0.65 in both the discovery and validation datasets, with the top performer CC16 having an AUC of 0.81 in the discovery dataset (Table 3). Gene set enrichment showed CC13 as nucleoside triphosphate metabolic process and cell envelope, CC16 as cell cycle and cellular response to DNA damage and stress, CC17 as DNA packaging complex.

**Table 3:** Canonical components (CCs) with AUC >0.65 in both the discovery and validation cohorts, based on a *p*-value < 0.05

Canonical Component	Discovery AUC	Validation Auc	GO Terms
CC 13	0.792	0.667	ORGANELLE INNER MEMBRANE
			MITOCHONDRIAL ENVELOPE
			MITOCHONDRIAL PART
			ENVELOPE
			NUCLEOSIDE TRIPHOSPHATE METABOLIC PROCESS
CC 16	0.818	0.667	CELL CYCLE
			CELLULAR RESPONSE TO DNA DAMAGE STIMULUS
			CELLULAR RESPONSE TO STRESS
			CELL CYCLE PROCESS
CC 17	0.766	0.667	GO_DNA_PACKAGING_COMPLEX
			GO_PROTEIN_DNA_COMPLEX

### Molecular subtype analysis

All 26 patients were classified using the following molecular subtyping: Baylor, UNC, CIT, Lund, MD Anderson, TCGA, and Consensus Class [13, 17–20, 22]. The Baylor classification is divided into basal and differentiated. The UNC classification is split into basal-like and luminal. The CIT classification has MC1-7, while the Lund subtype breaks down into: urobasal A, genomically unstable, urobasal B, squamous cell carcinoma like, and infiltrated. MD Anderson (MDA) is divided into luminal, basal and p53-like. The TCGA has 5 subtypes: luminal infiltrated, luminal papillary, luminal, basal squamous, and neuronal. Lastly, the Consensus Classification was an international effort that reconciled six previously mentioned classification scheme into a new consensus classification that was divided into six subtypes: luminal papillary, luminal nonspecified, luminal unstable, stroma-rich, basal/squamous, and neuroendocrine-like.

Table 4 includes the subtype classification for each patient using the aforementioned 6 different molecular classification schemes. For the discovery cohort, 6 out of 11 NR were p53-like (MDA), which is known to be a chemo-resistant phenotype. In addition, the majority of the six patients who were classified as p53-like (MDA) in the NR discovery group were also defined as basal (UNC), MC4 (CIT), and stroma-rich (Consensus Class). Of the remaining patients in the NR discovery cohort, 3 were defined as the MDA basal subtype and 2 were defined as the luminal subtype. The R discovery cohort had one chemotherapy resistant p53-like patient (MDA), while the remaining 6 patients were evenly split between the MDA basal and luminal subtypes.

**Table 4:** Patient molecular subtype classifications, as defined by the baylor, UNC, CIT, lund, MD anderson, TCGA, and consensus class classifications

Discovery Cohort							
Path Status	Baylor	UNC	CIT	Lund	MDA	TCGA	Consensus Class
NR1	Basal	Basal	MC4	Ba/Sq-Inf	p53-like	Luminal_infiltrated	Stroma-rich
NR2	Basal	Basal	MC4	Mes-like	p53-like	Basal_squamous	Stroma-rich
NR3	Differentiated	Luminal	MC4	GU	p53-like	Luminal_infiltrated	LumU
NR4	Basal	Basal	MC4	Mes-like	p53-like	Luminal_infiltrated	Stroma-rich
NR5	Basal	Basal	MC4	Mes-like	p53-like	Basal_squamous	Stroma-rich
NR6	Differentiated	Luminal	MC4	UroA-Prog	p53-like	Luminal_infiltrated	LumP
NR7	Basal	Basal	MC4	GU-Inf	basal	Luminal_infiltrated	Ba/Sq
NR8	Differentiated	Luminal	MC1	UroA-Prog	luminal	Luminal_papillary	LumP
NR9	Basal	Basal	MC7	Ba/Sq-Inf	basal	Basal_squamous	Ba/Sq
NR10	Basal	Basal	MC7	Ba/Sq	basal	Basal_squamous	Ba/Sq
NR11	Differentiated	Basal	MC7	UroB	luminal	Basal_squamous	Ba/Sq
R1	Differentiated	Basal	MC4	GU-Inf	p53-like	Luminal_infiltrated	Stroma-rich
R2	Basal	Basal	MC7	Mes-like	basal	Basal_squamous	Ba/Sq
R3	Basal	Basal	MC7	Ba/Sq	basal	Basal_squamous	Ba/Sq
R4	Basal	Basal	MC7	UroB	basal	Basal_squamous	Ba/Sq
R5	Differentiated	Luminal	MC1	GU	luminal	Luminal_papillary	LumU
R6	Differentiated	Luminal	MC1	UroA-Prog	luminal	Luminal	LumP
R7	Differentiated	Luminal	MC1	UroA-Prog	luminal	Luminal_infiltrated	LumP
**Validation Cohort**							
NR12	Differentiated	Basal	MC4	Uro-Inf	p53-like	Luminal_infiltrated	Stroma-rich
NR13	Differentiated	Basal	MC4	UroC	p53-like	Luminal_infiltrated	Stroma-rich
NR14	Differentiated	Basal	MC4	Mes-like	p53-like	Luminal_infiltrated	Stroma-rich
NR15	Basal	Basal	MC7	Ba/Sq-Inf	basal	Basal_squamous	Ba/Sq
NR16	Differentiated	Luminal	MC1	UroC	luminal	Luminal_papillary	LumU
R8	Differentiated	Luminal	MC1	UroC	luminal	Luminal_infiltrated	LumU
R9	Basal	Basal	MC7	Ba/Sq-Inf	basal	Basal_squamous	Ba/Sq
R10	Differentiated	Luminal	MC3	GU	luminal	Luminal	LumU

For the validation cohort, 3 out of the 5 NR patients were p53-like (MD Anderson), as well as differentiated (Baylor), basal (UNC), MC4 (CIT), luminal-infiltrated (TCGA), and stroma-rich (Consensus Class). The remaining 2 patients in the NR validation cohort were split between luminal and basal (MDA), while of the three patients in the R validation, 2 were defined as MDA luminal and 1 as basal.

When looking at NR for both discovery and validation combined, subtypes that correlated the most with being a NR include: basal (Baylor) 63% (7/11), differentiated (Baylor) 57% (8/14), basal (UNC) 68% (11/16), MC4 (CIT) 90% (10/11), p53-like (MDA) 90% (9/10), luminal-infiltrated (TCGA) 72% (8/11), basal-squamous (TCGA) 60% (6/10) and stroma rich (Consensus Class) 100% (8/8). Overall, the NRs from both cohorts have a significant portion of patients who are MC4 (CIT), p53-like (MD Anderson), and stroma-rich (Consensus Class). When examining the known chemotherapy resistant p53-like subtype within our entire cohort more closely, using Fisher’s exact test with a 2 × 2 contingency table (9 p53-like NRs and 1 p53-like R versus 7 non p53-like NRs and 9 non p53-like Rs), shows the p53-like subtype is significantly associated with being a NR (*p*-value of .03674, Odds Ratio 10.5). Lastly, for both the R discovery and validation cohorts, the subtypes for each classification were mixed without any definitive subtype making up a significant majority for each molecular classification.

## DISCUSSION

The preceding analysis combines differential mRNA and miRNA expression in known MIBC phenotypes to better understand the molecular driver behind NAC response. The majority of molecular classification and subtyping to date has been performed with variations in gene expression technology, cohorts that have varied in size, and has primarily relied on unsupervised hierarchical clustering without factoring in NAC response. We report significant gene sets associated with NAC response phenotype, as well as three multigene and miRNA signatures generated by CCA that can be used to potentially classify NAC response.

To our knowledge, our study is the first to use combined differential mRNA and miRNA expression in MIBC to identify a NAC response signature. The mitochondrial and metabolic signature seen in CC13, the cell cycle signature and response to DNA damage seen in CC16, and the DNA packaging signature in CC17 all appear to be driven by the responders in the discovery cohort given the overlap seen when comparing these CC signatures to the GSEA results for this cohort (i.e., Gene Ontology Biological Process (GOBP)_mitochondrial gene expression, GOBP_DNA replication initiation, GOBP_DNA Unwinding involved in DNA Replication, seen in Supplementary Table 3).

Biologically, these CC and GOBP signatures make sense given MIBC’s historical success with cisplatin based chemotherapy regimens, in which cisplatin causes DNA damage, blocks cell division and triggers apoptotic cell death [24]. When looking at GOBP_mitochondrial gene expression, it is known that mitochondria take part in short and long patch base excision repair, which is achieved by DNA polymerase γ. This is critical for the DNA repair pathway to maintain whole genome stability, as well as being a primary source for reactive oxygen species (ROS) and regulating apoptosis [25, 26]. Within this pathway, several mammalian mitochondrial ribosomal small subunit (MRPS) genes are upregulated in the R discovery cohort, including MRPS12, MRPS34, MRPS28, MRPS14, and MRPS2. Upregulation of these genes *in vitro* may restore chemosensitivity in resistant bladder cancer cell lines, potentially by restoring the cells ability to undergo apoptosis.

Another intriguing pathway identified in the R discovery cohort was GOBP_Sulfur Compound Catabolic Process. Hydrogen sulfide inhibition in one study was shown to enhance cisplatin induced apoptosis both *in vitro* and *in vivo,* and exogenous administration in another study enhanced *in vitro* cell proliferation [27, 28]. Overall, there appears to be a metabolic shift in the R discovery cohort that likely contributed to chemosensitivity, as evident by other significant pathways expressed: GOBP_Glucosamine Containing Compound Metabolic Process, GOBP_Threonine Type Peptidase Activity, GOBP Isoprenoid Biosynthetic Process, and GOBP_ Cellular Response to pH.

When further examining GOBP_DNA replication initiation and GOBP_DNA Unwinding involved in DNA Replication , the minichromosome maintenance complex (MCM) genes play a crucial role in both DNA replication and unwinding. MCM2-7 forms a helicase complex that contributes to both the initiation and elongation phase of DNA replication [29]. MCM2-3 and 5–6 are significantly upregulated in the R discovery cohort. Although the knockdown of MCM genes has been linked to decreased cellular proliferation [30], increased expression could theoretically promote cells to undergo ineffective DNA replication due to platinum DNA intercalation that eventually leads to cell death. XPA is also substantially enhanced in the NR discovery cohort. It is a zinc finger protein that plays a central role in nucleotide excision repair (NER), which is responsible for repair of DNA adducts induced by cisplatin chemotherapy. The XPA protein binds to DNA and several NER proteins, acting as a scaffold to assemble the NER incision complex at sites of DNA damage [31]. Targeting MCM and XPA genes *in vitro* may be another potential candidate for inducing chemotherapy response.

Expanding upon the previous two pathways, DNA packaging plays a critical role in many nucleic acid processes, including transcription, DNA repair, and DNA replication. When DNA damage is induced by cisplatin, chromatin encounters dramatic changes in response to DNA damage both locally and genome-wide, which initiates the DNA repair process [32]. Genes implicated in the DNA packaging pathway may potentially change cisplatin response through change in their expression. ELK4 is markedly upregulated in the NR discovery cohort. This gene is a member of the Ets family of transcription factors, whose functions are DNA-binding transcription factor activity and chromatin binding [33]. FOXA3 is dramatically increased in the R discovery and validation cohort. This gene is a member of the forkhead class of DNA-binding proteins and also interacts with chromatin [34]. These chromatin interactive genes involved in DNA packaging will be further studied to predict cisplatin response.

Although no miRNA had statistically significant differential expression in the discovery cohort, several miRNAs significantly contribute to the three relevant CC signatures. The following miRNAs listed for each CC may be potential candidates for modulating chemotherapy sensitivity or serve as therapeutic targets. For CC13, miR-192 and mi-194 overexpression has been shown to inhibit cell proliferation in bladder cancer cells *in vitro* [35, 36]. In CC16, miR-15 has been shown to inhibit bladder cancer cell proliferation, migration and invasion *in vitro* by targeting BMI1 through the PI3K/AKT pathway [37]. Also, one study found that miR-34 upregulated PTEN and inhibited bladder cancer cell migration and invasion [38]. For CC17, miR-455-5p has been shown to induce cisplatin resistance in bladder cancer cells by regulating Notch1 [39].

Gene expression profiling of MIBC holds promise for future individualization of therapy, and several studies have attempted to predict chemosensitivity using the molecular profile of tumors. Two studies using whole-exome-sequencing and DNA sequencing targeting 287 cancer-related genes respectively, found that alterations in DNA repair genes may correlate with response to chemotherapy: ERCC2, ATM, RB1, and FANCC [11, 12]. Of those 4 genes, FANCC was the only one found to have significant expression, albeit in our NR discovery cohort.

Lastly, of note, two studies used microarray analysis of TURBTs to create a prediction scoring system that can potentially determine response to GC or MVAC NAC, respectively [40, 41]. Although these two studies created their prediction scoring system based on known NAC response, responders in these studies were defined as having had at least a partial response (either >60% tumor shrinkage or ≤pT1 after 2 cycles of chemotherapy) unlike our cohort where the majority of the responders were pT0 (we included 1 pTis) and response was determined after 4 cycles of chemotherapy (standard of care) [42]. Interestingly enough, 2 of the genes used in the prediction scoring system developed by Kato et al. had concordant significant expression in our discovery cohort: Spry1 was increased in NR and CRKL increased in R. PNPO was increased in our R discovery cohort, while it was increased in the NR cohort form Kato et al. [40]. None of the genes from the Takata et al. scoring system had significant expression in our discovery cohort.

In addition to the aforementioned molecular analysis, subtyping patients based on molecular expression is another promising approach that has potential for determining chemosensitivity. However, the TCGA and several other studies have identified molecular expression subtypes using hierarchical classification techniques that to date have served more as biological classifications rather than predictors of clinical outcome [13, 17–22]. In the TCGA analysis, predicted chemotherapy response is hypothesis driven from patients with incomplete or unknown treatment history, and not derived from comparing molecular phenotypes between NAC responders and non-responders. The basal/squamous subtype is predicted to be the most chemotherapy sensitive, with luminal-infiltrated and luminal-papillary having lower response rates to NAC. However in the NR discovery cohort, 45% (5/11) were basal/squamous and 42% (3/7) of the R discovery cohort were either luminal infiltrated or luminal-papillary. Despite there being some basal/squamous patients in the NR discovery cohort, basal expression does appear to play a significant role in chemotherapy response as shown by the top two GSEA pathways in the R discovery cohort: GOBP_Keratinization and GOBP_Keratinocyte Differentiation. Significant genes within these two pathways are another candidate for further exploration to better characterize chemotherapy response.

Expanding upon established subtyping analysis, Seiler et al. used 149 genes from the subtypes identified by hierarchical clustering from four studies (TCGA, Lund, MDA, UNC), to develop a single-sample genomic subtyping classifier (GSC) that showed patient outcomes after NAC varies by four molecular subtypes: basal, claudin-low, luminal, or luminal infiltrated subtype [23]. The basal subtype benefited most from NAC, whereas the other three subtypes did not demonstrate a significant survival advantage with NAC. Although the four subtypes are associated with overall survival predictions after NAC, the subtypes could not establish which patients would have a pathological response to NAC. A lack of correlation between NAC pathological response and overall survival is a limitation that conflicts with previously mentioned studies, although the study may have been underpowered to find this correlation.

In contrast to the TCGA and Seiler et al. analysis, Choi et al. in their initial discovery cohort and in a subsequent phase 2 follow-up study, found that p53-like tumors were mostly resistant to NAC [21, 43]. 54% (6/11) of our NR discovery cohort were classified as p53-like, and when looking at all of the patients classified as p53-like in both the discovery and validation cohorts combined, 90% (9/10) were classified as NR. This correlation (being classified as p53-like and being a NR) was statistically significant in our cohort. Overall, this finding strengthens both the validity of the Choi et al. p53-like subtype and our own molecular expression analysis. Similar to what Choi et al. reported, the MDA basal and luminal subtypes for our two cohorts are mixed between NR and R. One patient in the R discovery cohort was defined as p53-like, which raises the point that relying on one classification system may have limited potential. However, given that MC4 (CIT) and stroma rich (Consensus Class) were also highly correlated with a NR phenotype, tumors that express these 2 subtype classifications, as well as p53-like may have the potential to be confidently classified as a NR. This may be worth assessing and validating in a larger follow-up study.

Limitations of our study include a small patient size in both the discovery and validation cohorts, as well as intratumoral heterogeneity that may limit reproducibility of results and subtype analysis. However, the overlap of a known chemoresistant molecular subtype (MDA’s p53-like subtype) and our cohort of NR, gives validity to our small sample size. Furthermore, CCA accounts for intratumor heterogeneity when creating *de novo* mRNA and miRNA modules across different samples and in effect theoretically limits heterogeneity. Another limitation is the decreased number of significant differentially expressed genes in the validation cohort as compared to the discovery cohort. We suspect that this is largely due to the fewer number of patients in the validation cohort.

In conclusion, our results identify molecular signatures that can be used to differentiate MIBC NAC responders versus non-responders. We have presented the salient molecular pathways and relevant genes, including mitochondrial response gene expression (MRPS12, MRPS34, MRPS28, MRPS14, and MRPS2), DNA replication initiation, and DNA unwinding and DNA damage (MCM2-3, MCM5-6 and XAP , ELK4, and FOXA3) that can be further analyzed to better understand NAC response. The above mentioned genes derived from their respective three pathways may be selected as part of a NAC response biomarker panel. In addition, we have highlighted the utility of molecular subtyping in relation to NAC response. If validated in a larger cohort, these findings may help deliver chemotherapy to those patients most likely to respond.

## MATERIALS AND METHODS

### Patient selection

A total of 26 patients with known NAC response were identified, and inclusion in either the discovery or validation cohort was chosen at random. The discovery cohort consisted of 7 NAC responders and 11 non-responders, while the validation cohort consisted of 3 responders and 5 non-responders. The Northwell Health System Institutional Review Board and Regional Ethics Committee granted research approval for the study with waivers of HIPAA authorization and informed consent. Clinical investigation was conducted according to the principles outlined in the Declaration of Helsinki. TURBT specimens from the Northwell Health pathology department were received as formalin-fixed, paraffin-embedded (FFPE) tissue blocks. Pathologic response at the time of cystectomy was determined using the American Joint Committee on Cancer staging [44].

### RNA extraction

A genitourinary oncology pathologist at Northwell Health reviewed all FFPE tissue blocks and their corresponding H&E slides, outlining neoplastic tissue on the H&E slide. Tissue blocks were matched up with their respective slides and approximately 35 mg of pre-identified tumor was cut away from the blocks using a scalpel. RNA was extracted from FFPE tissue using the RecoverAll™ Total Nucleic Acid Isolation Kit with quality control performed using an AB Bioanalyzer.

### mRNA expression analysis

RNA was sequenced using the Illumina TruSeq RNA Access Library Prep Guide and NextSeq 500. Sequenced segments were aligned using the STAR2 aligner [45]. Gene counts were assessed using ht-seq counts [46]. Differential expression was calculated using DESeq2 [47]. Adjustment for multiple corrections was performed using Benjamini-Hochberg method. Pathway enrichment analysis was performed using GSEA [48, 49].

### miRNA expression analysis

Differential expression of 754 miRNAs was analyzed using the TaqMan Open Array miRNA qPCR panel. A Ct threshold of 30 was used and miRNAs were filtered by removing miRNAs detected in <10% of samples. The top 10 most stable miRNAs from the NormPCR package were used as housekeeping miRNAs, which were used to normalize raw Ct values with deltaCt normalization [50, 51]. The limma package was used to calculate differential expression [52].

### Canonical correlation analysis and validation

Sparse canonical correlation analysis was used to identify *de novo* mRNA and miRNA Canonical Components (CCs) with AUC to NAC response was determined using pROC [53, 54]. Data analysis was performed using R and tidyverse [55, 56]. Gene Ontology (GO) enrichment was performed using a Fisher’s test.

### Subtype analysis

Subtyping was performed using the BCLAsubtyping R package created by Kamoun et al. [57].

## SUPPLEMENTARY MATERIALS















## Abbreviations

MIBC: muscle invasive bladder cancer
NAC: neoadjuvant chemotherapy
mRNA: messenger RNA
miRNA: microRNA
NCI-DTP: National Cancer Institute’s Developmental Therapeutics Program
COXEN: Coexpression Extrapolation
R: responders
NR: non-responder
TURBT: transurethral resection of bladder tumor
GC: gemcitabine cisplatin
ddMVAC: dose-dense methotrexate vincristine adriamycin, cisplatin
CCA: canonical correlation analysis
CCs: canonical components
PCA: Principal Component Analysis
GSEA: gene set enrichment analysis
MDA: MD Anderson
GOBP: Gene Ontology Biological Process
ROS: reactive oxygen species
MRPS: mitochondrial ribosomal small subunit
MCM: minichromosome maintenance complex
NER: nucleotide excision repair
GSC: genomic subtyping classifier
