# Supplementary material for: Predictive molecular biomarkers for determining neoadjuvant chemosensitivity in muscle invasive bladder cancer
Source: Oncotarget. 2022 Nov 2;13:1188–200. doi: 10.18632/oncotarget.28302 (PMC9629806; doi:10.18632/oncotarget.28302)
Supplement: Supplementary file 1 [file oncotarget-13-28302-s001.pdf]

Predictive molecular biomarkers for determining neoadjuvant chemosensitivity in muscle invasive bladder cancer

SUPPLEMENTARY MATERIALS

Supplementary Table 1: Significant genes in discovery cohort. See Supplementary Table 1

Supplementary Table 2: Significant genes in validation cohort. See Supplementary Table 2

Supplementary Table 3: GSEA. See Supplementary Table 3

Supplementary Table 4: Significant miRNA in discovery cohort. See Supplementary Table 4

Supplementary Table 5: Significant miRNA in validation cohort. See Supplementary Table 5

Supplementary Table 6: CC analaysis with CC genes and miRNA and GO terms. See Supplementary Table 6
